# Evaluation of the humoral immune response induced by vaccination for canine distemper and parvovirus: a pilot study

**DOI:** 10.1186/s12917-018-1673-z

**Published:** 2018-11-16

**Authors:** Beatriz Vila Nova, Eva Cunha, Nuno Sepúlveda, Manuela Oliveira, Berta São Braz, Luis Tavares, Virgílio Almeida, Solange Gil

**Affiliations:** 1 Faculty of Veterinary Medicine, University of Lisbon, Av. Universidade Técnica, 1300-477 Lisbon, Portugal; 2 CIISA- Centre for Interdisciplinary Research in Animal Health, Faculty of Veterinary Medicine, University of Lisbon, Av. Universidade Técnica, 1300-477 Lisbon, Portugal; 3 London School of Hygiene and Tropical Medicine, London, UK; 4 Centro de Estatística e Aplicações da Universidade de Lisboa, Lisbon, Portugal

**Keywords:** CDV, CPV, Vaccination, Humoral immunity, Maternal antibodies, Seroprevalence, Seroreversion

## Abstract

**Background:**

Canine Distemper Virus (CDV) and Canine Parvovirus (CPV) lead to infections with high mortality rates in dogs. These viruses affect unvaccinated dogs or dogs with incomplete vaccination protocols. Vaccination plays an important role in reducing death rates, preventing clinical cases and controlling the spread of virus However, the efficacy of vaccination might be affected by different factors including vaccine scheduling and the neutralization of the vaccine targets by maternal antibodies. In face of these factors, the main goals of this study are (i) to investigate the antibody responses of puppies undergoing different primary vaccination protocols against CPV and CDV and (ii) to estimate the time until seroreversion in adult dogs unvaccinated for at least 3 years.

**Results:**

Antibody protection against CDV and CPV was evaluated in a total of 20 dogs: 5 puppies that initiated immunization at 6 weeks after birth (group A), 8 animals that started vaccination between 8 and 12 weeks of age (group B), and 7 adult dogs that have not been vaccinated for at least 3 years (group C). Blood samples were collected from each animal, with 3 to 4 weeks apart. Antibody responses were measured using indirect ELISA. In the second immunization point, no significant differences were found between the seroconversion of groups A and B for each viral infection (*p* = 0.81 and 0.20 for CDV and CPV, respectively). In the third immunization, there was evidence for a shorter time to achieve a protective titer against CPV in group B when compared to group A (*p* = 0.015). Similar evidence was not found for CDV (*p*-value = 0.41). In Group C, the average time until seroveversion was estimated at 2.86 years and 7.63 years for CDV and CPV, respectively.

**Conclusion:**

Vaccine response to CDV and CPV is specific in each individual. Effective immune protection in primary vaccination depends mainly on the initial titer of maternal antibodies acquired by the neonate. Other factors such as environmental exposure, immunization schedules and immune system activity influence the duration of immunity in adult dogs. The variability found reinforces the need to determine individual humoral immunity levels in order to assess vaccine efficacy.

## Background

Canine parvovirus (CPV) and Canine Distemper Virus (CDV) cause life-threatening infections with a major impact on the canine population [1, 2]. These viruses are distributed worldwide affecting both domestic and wildlife mammals [2]. In dogs, CPV is an important enteric pathogen because of the associated high morbidity and mortality rates [3]. It can affect dogs of any age, but severe infections are most likely to occur between 6 weeks and 6 months after birth [3]. Since CPV is a DNA virus without an envelope, it is highly resistant to adverse environmental conditions and persistent on fomites and cage floors for more than a year [4]. CPV inactivation can be achieved by agents include sodium hypochlorite and potassium peroxymonosulfate [4, 5].

Infections by CDV show a range of symptoms in dogs including fever, respiratory, enteric and neurological signs [6, 7]. In these infections, young dogs with age between 3 and 6 months are more like to show clinical symptoms than older ones [6]. In contrast to CPV, CDV can be easily inactivated using UV radiation, heat, desiccation, oxidizing agents, detergents and lipid solvents [6].

Vaccination has proven successful to prevent future CPV and CDV infections [8–10]. Current recommendations suggest that puppies should have the first (or primary) vaccination scheduled between the first 6 and 8 weeks of age, followed by several boosts administered in intervals of 3 or 4 weeks until 16 weeks of age. At this point, maternal antibodies are expected to have declined so their chance of interfering with the vaccine antigen is low [11–13]. The number of vaccine boosts required during this phase might vary with age at which the vaccination protocol began [10]. For example, if the first vaccination has been administered 16 weeks after birth, a single dose may be sufficient to achieve protection [10]. In adult dogs, vaccination should be carried out every 3 years [10].

Vaccination stimulates both humoral response via antibody production and cellular responses via B and T lymphocytes [14]. The duration of immunity is mainly dependent on the degree of immunological memory developed [12, 14, 15]. However, it is unclear whether a vaccinated dog is fully protected throughout its life or whether revaccination is always necessary [16]. To clarify these issues, it is important to quantify the rate by which vaccinated animals become sero-naive again, the so-called seroreversion rate (SRR) [14]. Under the assumption that serum antibody titer is correlated with protective immunity, antibody quantification may be seen as a way to assess the immune protection in vaccinated and unvaccinated animal [17, 18].

The pharmaceutical industry has been developing tests for antibody quantification associated with CPV and CDV exposure in canines [19]. These tests should be performed within 3 to 4 weeks after vaccine administration to assess the necessity of further vaccination steps [10]. This evaluation is essential to reduce the number of necessary immunization shots and to detect possible vaccine failures, thus, decreasing the chance of having a large pool of susceptible animals in the population [20].

From an animal health perspective, the improvement of current vaccine protocols is important to reduce the high incidence of CPV and CDV in the canine population. In this scenario, this work has the objective of investigating the seroconversion to CPV and CDV in two groups of puppies undergoing two different primary vaccination protocols. It also aims to evaluate the serological status associated with CPV and CDV in a group of adult dogs unvaccinated for at least 3 years and to estimate the respective seroreversion rate.

## Results

All dogs included in this study were dewormed and evaluated by a veterinarian from the Veterinary Teaching Hospital (VTH, Faculty of Veterinary Medicine, University of Lisbon) before each vaccination. Only dogs deemed healthy were allowed to start the vaccination protocol. Dogs deemed unhealthy at the vaccination appointment, dogs that failed the immunization schedule, dogs that started vaccination after 12 weeks of age and dogs whose age was unknown, were excluded from the study.

The above exclusion criteria discarded 64 out of 84 available dogs of different races, sex and ages. The study was then carried out in 20 dogs with following distribution across three vaccination groups: A (*n* = 5), B (*n* = 8) and C (*n* = 7) (Tables 1 and 2).

**Table 1:** Ages of dogs in group A and B at the time of sample collection and vaccination. Three blood samples were collected from each animal at the time of vaccination

GROUP	DOG (ID)	Age (weeks) 1st sample	Age (weeks) 2nd sample	Age (weeks) 3rd sample
A	A1	6,3	9,3	10,7
	A2	6,3	9,3	10,7
	A3	6,3	9,3	10,7
	A4	6,3	9,3	10,7
	A5	6,3	9,3	10,7
B	B1	8,9	11,9	15,6
	B2	8,7	13,1	20,7
	B3	11,7	15,9	19,9
	B4	8,9	13,3	16,7
	B5	11,1	15,4	21,4
	B6	11,1	15,4	21,4
	B7	9,6	13,9	18,9
	B8	8	12	16

**Table 2:** Ages of dogs and time elapsed since last vaccination in group C at the time of sample collection

Dog (ID)	Age (years)	Time elapsed since last vaccination (in years)
C1	3,7	3
C2	7	5
C3	6	6
C4	18	8
C5	9	9
C6	16	12
C7	8	Never vaccinated

Group A consisted of five dogs from the same litter that started vaccination at 6 weeks and were followed for humoral protection evaluation until 12 weeks of age (Table 1). Group B encompassed eight older puppies from different origins, which began vaccination at 8 to 12 weeks. This group enabled the analysis of antibody protection beyond the 16 weeks of age. In these groups, there was no data on immune status of the respective parents. It was also unknown the amount of colostrum ingested by each puppy. Group C referred to seven adult animals unvaccinated for at least 3 years (Table 2). The age of these animals varied from 3.7 to 18 years old with an average of 9.7 years. Six out of seven dogs were vaccinated before this study. The average time since vaccination was 7.2 years with a range from 3 to 12 years.

In order to assess humoral response/seroconversion, blood samples were collected before and after vaccination in groups A and B. There was then a total of 39 blood samples. Antibody quantification was performed using indirect ELISA [10]. In Group C, a single blood sample per dog was collected for serological screening.

**Fig. 1:**
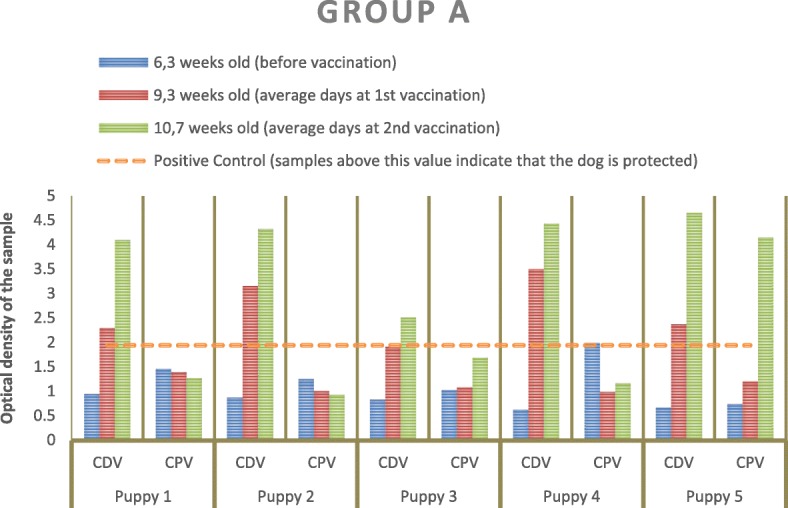
Results for CDV and CPV after measurement of the optical densities of the group A samples in the spectrophotometer. The dashed line corresponds to the optical density of the positive control so that all samples above the dashed line are positive

In group A, all dogs were unprotected against CDV before vaccination using TiterCHEK titres less than 1:16 (TITERCHEK® CDV-CPV, Zoetis). Three weeks after the first immunization (9.3 weeks), 80% of the dogs already showed a protective CDV SN titer and this percentage increased to 100% after the second vaccination (10.7 weeks) (Fig. 1).

With respect to CPV, there was a single animal (20%) who was protected before vaccination. This animal showed a TiterCHEK titre equivalent to a 1:80 (TITERCHEK® CDV-CPV, Zoetis). After the first vaccination at 9.3 weeks, none of the animals were protected and, after the second vaccination (10.7 weeks), only one dog (20%; one out of five) developed protection (Fig. 1). The remaining 80% of the puppies were still unprotected after the administration of two vaccine doses.

**Fig. 2:**
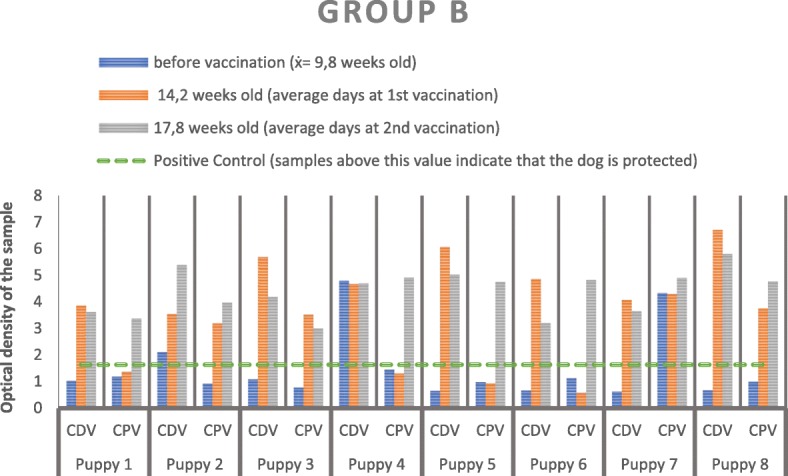
Results for CDV and CPV after measurement of the optical densities of the group B samples in the spectrophotometer. The dashed line corresponds to the optical density of the positive control so that all samples above the dashed line are positive

In group B, two animals (B4 and B7) received the first vaccine 3 weeks before the collection of first blood sample. On average the first sample was obtained at 9.8 weeks after birth, the second at 14.2 and the third at 17.8. In this group, CDV evaluation before vaccination revealed a protective antibody level in two out of eight dogs. However, one dog (B4) had been previously vaccinated. After the first vaccination, all dogs showed CDV protection with higher values than the protection baseline, which was maintained after the second vaccination (Fig. 2).

Concerning CPV, one dog showed protective HI titers before vaccination (B7). However, after the first vaccination, 50% (4/8) of the animals revealed HI titers higher than 1:80. After the second vaccination, all animals had protective titers against this virus (Fig. 2).

In the second immunization point, no significant differences were found between the seroconversion of groups A and B for each viral infection (*p* = 0.81 and 0.20 for CDV and CPV, respectively). However, in the third immunization point, there was evidence for higher seroconversion against CPV in group B (*p* = 0.015). This result suggested a shorter time to achieve a protective titer against CPV in this group when compared to group A (*p* = 0.015). Similar evidence was not found for CDV (*p*-value = 0.41). Both groups appeared then to require the same amount of time to acquire immunity against this virus.

**Fig. 3:**
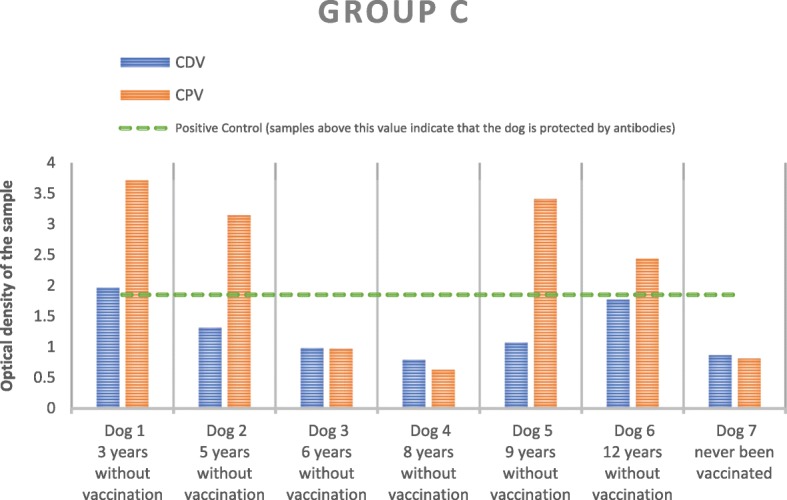
Results for CDV and CPV after measurement of the optical densities of the group C samples in the spectrophotometer. The dashed line corresponds to the optical density of the positive control so that all samples above the dashed line are positive

In Group C, there was only one animal (14.3%) that was protected against both viruses (Fig. 3). Three dogs of this group (C2, C5 and C6) had protective titers against CPV only. The unvaccinated dog n° 7 was unprotected against both virus as well as dogs C3 and C4. Overall, 57 and 14% of dogs were protected against CPV and CDV, respectively. Using data of the vaccinated animals from this group, SRR was estimated at 0.350 for CDV. This estimate predicted an average time of 2.86 years to seroreversion. The respective seroconversion rate (SCR) was estimated at zero, a value consistent with the lack of information to estimate natural exposure to that virus in vaccinated animals. With respect to CPV, the estimate of SRR was 0.131, which implied an average of 7.63 years to seroreversion. In this case, the seroconversion rate is estimated at 0.21, a value associated with an average of 4.76 years between infections.

## Discussion

Vaccination is usually seen as part of a comprehensive health program for both humans and animals in the fight against infectious diseases and in the prevention of future outbreaks [14]. To achieve measurable impacts in the population, immunization schedules should be optimized according to the specific context of each animal, including its species, age, habitat, lifestyle, effectiveness of available vaccines and the underlying disease prevalence [10, 13].

In group A, only one dog had a protective titer for CPV prior to vaccination. This result was consistent with a persistence of maternal antibodies in this animal. After the second vaccination, only puppy n° 5 was protected for CPV. Regarding this dog, the vaccine protocol for CDV and CPV could have ended at 10 weeks of age. In contrast, almost all dogs were antibody-protected against CDV after the first vaccination. The variability of the maternal antibodies level may be explained by the amount of colostrum ingested by the puppies and by the mother’s antibodies titer [11, 13, 17]. In this case, the titer was the same for all puppies and therefore the amount of colostrum ingested by each puppy might be the only variable to consider. Therefore, the only protected puppy against CPV in the first assessment probably ingested more colostrum. It is likely that the maternal antibodies titer was low for CDV, not allowing for an effective transfer of antibodies through colostrum. Despite the fact that only one puppy was protected against CPV, none of the puppies responded to the first CPV immunization. These puppies probably had maternal antibodies against CPV higher than 1:20, which hindered vaccination success [11].

Group B consisted of slightly older dogs, which started the vaccination protocol after 8 weeks of age. All dogs in this group developed an immune response to CDV after the first vaccine administration. Four weeks after the second administration, which matched with more than 16 weeks of age, all puppies were protected against both CDV and CPV, thus, ending primary vaccination protocol successfully. This suggests a low level of maternal antibodies at the start of the vaccination protocol, which were insufficient to neutralize the vaccine antigen at this age. With respect to CDV, there were no changes between the results obtained after the first and the second immunizations, revealing that only one vaccine administration could have been enough to protect these puppies against CDV infection. Regarding CPV, a single puppy showed a protective titer (higher than 1:80) before beginning the vaccination protocol, revealing a previous effective immune response, contrary to the other puppy, which also had been submitted to a previous vaccination. This variation may be explained by differences in maternal antibody titers, vaccine type, immune competence, environmental factors or even by individual biological differences [21]. Animals from group B would appear to achieve immunity against CPV faster than the group A. Similar evidence was not found for CDV.

In group C, only one vaccinated dog was protected against both viruses (Table 2 and Fig. 3). Three other vaccinated dogs had a protective humoral immunity against CPV. Yet, they did not present antibody protection against CDV. As previously described, CPV is ubiquitous and very resistant to adverse conditions and disinfectants. In contrast, CDV is extremely susceptible to environmental conditions [4–6]. This results in an increased natural exposure to CPV, leading to more frequent antibody boosts and a quicker development of immune protection when compared to the less-frequent CDV. In fact, the occasional exposure to CDV might not be sufficient to generate strong immune responses that, ultimately, lead to a protective antibody titer [22, 23]. This interpretation is in line with a zero estimation for SCR, a common proxy of exposure frequency. However, this group was very heterogeneous in terms of age, environmental exposure, immunization schedules and immune system activity [21]. Therefore, the respective results should be interpreted with caution. However, there is evidence of a long-term protective immunity induced by core viral vaccines in adult dogs that had not been revaccinated for more than 9 years [12, 14, 15]. In fact, our results suggest that the duration of antibody immunity against CPV is longer than the one against CDV in these adult dogs. Unfortunately, there was no information on the type of vaccines used and immunization schedules in order to understand this result better. Additional heterogeneity in antibody-induced immunity can also be derived from vaccines produced by different manufacturers, as demonstrated by a serological survey on 780 dogs vaccinated for CDV and CPV [23].

Overall, primary vaccination results for puppies showed that the antibody response occurred earlier to CDV vaccination than to CPV. These results suggest that during primary vaccination the response is individual and mostly dependent on the initial titer of maternal antibodies acquired by the neonate [11, 13]. Also, the use of multivalent vaccines may induce different immune responses against the immunogenic agents [24]. In some dogs, a single administration may be sufficient to provide protection, namely against CDV. Therefore, the use of monovalent vaccines may be advisable in individual cases rather than the administration of multivalent ones [24].

The variability found reinforces the importance of determining the antibody immunity of each animal [19]. ELISA tests provide an advantageous tool to confirm protection levels during consultation at a relatively low cost [19]. These tests should be primarily used to validate vaccine efficacy induced by primary vaccination, but also to assist the veterinarian in establishing dog-specific vaccination protocols [18, 20]. These tests may help to identify dogs susceptible to these infectious diseases as well as to reduce the frequency of necessary vaccination doses. As suggested by Twark & Dodds (2000), these tests should be implemented as a routine similarly to what occurs in the poultry and the pig industry [25]. This would lead to a better control of core viral diseases, such as those caused by CDV and CPV infections.

## Conclusion

Timely vaccination protocols are essential to prevent infectious diseases, such as those caused by CPV and CDV. Besides optimization of vaccination schedules, other factors including dogs’ age, protective antibody titer or even vaccine type should not be neglected in assessing the effectiveness of a given vaccine. In this scenario, the present study reinforces the evidence that antibody response to vaccination is specific to each animal. This variation supports the necessity of assessing individual humoral immunity before vaccination. This evaluation can be easily performed using ELISA assays during consultation at the veterinary clinic.

## Methods

All puppies from 6 to 12 weeks of age submitted to primary vaccination and adult dogs unvaccinated for at least 3 years, presented at VTH, were selected to this study. All dogs included in this study were dewormed and evaluated by a veterinarian prior to each vaccination and considered healthy and able to initiate the vaccine protocol. Animals were distributed by three groups: A – puppies which started the vaccine protocol at 6 weeks; B - puppies which started the vaccine protocol at 8 to 12 weeks; C - adult animals, unvaccinated for at least 3 years. Primary vaccination in group A was performed using the multivalent Nobivac® Puppy DP vaccine (MSD Animal Health Lda., Paço de Arcos, Portugal); for group B was used the multivalent Nobivac ® DHPPi vaccine (MSD Animal Health Lda., Paço de Arcos, Portugal).

Blood samples were collected (0.5 or 1 ml) from the lateral saphenous vein, prior to the administration of the first vaccine, and in the subsequent vaccine boosters.

Three blood samples were collected from each animal of groups A and B at the time of the vaccination consultations, 3 to 4 weeks apart. The first blood sample was collected before vaccination, allowing the evaluation of maternal antibodies in vaccine effectiveness. The remaining samples enabled the determination of the puppy’s immune response to vaccination. In adult dogs, which were unvaccinated for more than 3 years, only one blood sample was collected to investigate their humoral immunity status for CPV and CDV. Each sample was then centrifuged (6000 rpm for 10 min). Plasma was collected and stored in the freezer at − 20 °C until further use.

Unhealthy dogs, dogs that missed scheduled immunization appointments, or started vaccination after 12 weeks of age, or whose age was unknown were excluded from this study.

All procedures involving the manipulation of these dogs were performed after owner’s written consent and approval by the Committee for Ethics and Animal Welfare (CEBEA) of the Faculty of Veterinary Medicine – University of Lisbon, Portugal. All dogs that participated in this study were client-owned animals which joined the study during their current vaccination schedule protocol at VTH. All sampled animals stayed with their owners after each sample collection.

### Antibody evaluation

The antibody response was evaluated using a rapid test based on the indirect ELISA technique for antibody detection (TiterCHEK® CDV/CPV, Synbiotics Corporation, San Diego, USA). Results from samples were compared with the positive control provided, as well as with the negative control. For CDV, a positive result indicates a SN titer equal to or greater than 1:16, and a negative result a SN titer of less than 1:16 [20]. For CPV, a positive test result indicates a HI titer of 1:80 or higher, and a negative HI titer of less than 1:80 [20]. According to the test’s manufacturer, a comparative colorimetric evaluation by direct observation is sufficient to detect the positivity of a sample; however, this observation was further supported with a quantitative measurement of the absorbance on a spectrophotometer (FLUOstar optima BMT Labtech) to increase evaluation objectivity. A standard sample was used and evaluated at different wavelengths to determine which one should be applied. Readings were performed at 405 nm.

### Statistical analysis

The seroprevalences of groups A and B were compared with each other at the equivalent immunisation schedule using a Pearson’s chi-square test for two-way frequency table. To compare the induced immunizations against CDV and CPV, the respective data was transformed into a frequency vector (f1,f2,f3) where f1 is the frequency of animals in which transition from seronegative to seropositive (e.g., seroconversion) for CDV and CPV occurred in the same time interval, f2 is the frequency of animals in which seroconversion for CDV occurred earlier than for CPV, f3 is the frequency of animals in which seroconversion for CPV occurred earlier for CDV. It was then tested whether both viruses would induce the same time for seroconversion. This hypothesis was statistically translated into f2 = f3, or equivalently, f2/(f2 + f3) = 0.5. A Binomial test using the number of trials equal to f2 + f3 was used to formally test the hypothesis *p* = 0.5.

The statistical analysis of the group associated with adult dogs aimed to estimate the average time until a vaccinated animal becomes seronegative for each virus. With that purpose, a simple reversible catalytic model was fit to the corresponding data. In general, this model describes the stochastic transitions between seropositivity and seronegativity as function of age [26]. The respective transition rates are the so-called SCR and SRR assumed to be constant over time. The former rate is the frequency by which a seronegative animal becomes seropositive upon natural exposure to the infection, while the latter rate is the frequency by which a seropositive animal returns to a seronegative state in absence of recurrent exposure. In this study, it was assumed that (i) animals were all seropositive after vaccination, (ii) the time vaccination was the instant zero of the model, (iii) time since vaccination was the age variable of the model and (iv) serological outcomes of the individual were derived from independent Bernoulli trials. This model was then estimated using the maximum likelihood method, as described elsewhere [26]. Finally, for each virus, the average time to seroreversion was estimated as the inverse of the SRR estimate.

The whole statistical analysis was performed in the R software version 3.3.2. All statistical tests were carried out at 5% significance level. The sero-aid package was specifically used to fit the reversible catalytic model to the data. This package is available from NS upon request.

## Abbreviations

CDV: Canine Distemper Virus
CEBEA: Committee for Ethics and Animal Welfare (Faculty of Veterinary Medicine University of Lisbon)
CPV: Canine Parvovirus
HI: Hemagglutination Inhibition
SCR: Seroconversion rate
SN: Serum Neutralizing
SRR: Seroreversion rate
VTH: Veterinary Teaching Hospital

## References

[1] Greene CE, Decaro N, Greene CE (2012). Canine viral enteritis. Infectious diseases of the dog and cat.

[2] Greene CE, Levy JK, Greene CE (2012). Immunoprophylaxis. Infectious diseases of the dog and cat.

[3] Miranda C, Thompson GJ (2016). Canine parvovirus: the worldwide occurrence of antigenic variants. Gen Virol.

[4] Goddard A, Leisewitz A (2010). Canine Parvovirus. Vet Clin Small Anim.

[5] Crawford PC, Sellon RK, Ettinger SJ, Feldman EC (2010). Canine viral diseases. Veterinary internal medicine.

[6] Martella V, Elia G, Buonavoglia C (2008). Canine Distemper Virus. Vet Clin Small Anim..

[7] Loots A, Mitchell E, Dalton D, Kotzé A, Venter E (2017). Advances in canine distemper virus pathogenesis research: a wildlife perspective. J Gen Virol.

[8] Thiry E, Horzinek MC (2007). Vaccination guidelines: a bridge between official requirements and the daily use of vaccines. Rev Sci Tech.

[9] Greene CE, Vandevelde M, Greene CE (2012). Canine distemper. Infectious diseases of the dog and cat.

[10] Day MJ, Horzinek MC, Schultz RD, Squires RA (2016). WSAVA Guidelines for the vaccination of dogs and cats – compiled by the vaccination guidelines Group of the World Small Animal Veterinary Association. J Small Anim Pract.

[11] Pollock RV, Carmichael LE (1982). Maternally derived immunity to canine parvovirus infection: transfer, decline, and interference with vaccination. J Am Vet Med Assoc.

[12] Schultz RD, Thiel B, Mukhtar E, Sharp P, Larson LJ (2010). Age and long-term protective immunity in dogs and cats. J Comp Pathol.

[13] Mila H, Grellet A, Desario C, Feugier A, Decaro N, Buonavoglia C, Chastant- Maillard S (2014). Protection against canine parvovirus type 2 infection in puppies by colostrum-derived antibodies. J Nutr Sci.

[14] Day MJ, Day MJ (2012). Vaccination. Clinical immunology of the dog and cat.

[15] Jensen W, Totten J, Lappin M, Schultz R (2015). Use of serologic tests to predict resistance to canine distemper virus-induced disease in vaccinated dogs. J Vet Diagn Investig.

[16] Abdelmagid O, Larson L, Payne L, Tubbs A, Wasmoen T, Schultz R (2004). Evaluation of the efficacy and duration of immunity of a canine combination vaccine against virulent parvovirus, infectious canine hepatitis virus, and distemper virus experimental challenges. Vet Ther.

[17] Böhm M, Thompson H, Weir A, Hasted AM, Maxwell NS, Herrtage ME (2004). Serum antibody Titres to canine parvovirus, adenovirus and distemper virus in dogs in the UK which had not been vaccinated for at least three years. Vet Rec.

[18] Litster A, Pressler B, Volpe A, Dubovi E (2012). Accuracy of a point-of-care ELISA test kit for predicting the presence of protective canine parvovirus and canine distemper virus antibody concentrations in dogs. Vet J.

[19] Litster A, Nichols J, Volpe A (2012). Prevalence of positive antibody test results for canine parvovirus (CPV) and canine distemper virus (CDV) and response to modified live vaccination against CPV and CDV in dogs entering animal shelters. Vet Microbiol.

[20] Coyne MJ, Burr JH, Yule TD, Harding MJ, Tresnan DB, McGavin D (2001). Duration of immunity in dogs after vaccination or naturally acquired infection. Vet Rec..

[21] Tizard I, Ni Y (1998). Use of serologic testing to assess immune status of companion animals. J Am Vet Med Assoc.

[22] Olson P, Finnsdóttir H, Klingeborn B, Hedhammar A (1997). Short Communications: Duration of antibodies elicited by canine distemper virus. Vet Rec.

[23] Almendra C, Pinto O, Carmichael L, Tavares L (2005). Determinação dos níveis de imunidade humoral induzidos pela vacinação contra a Esgana e a Parvovirose caninas. RPCV.

[24] Wilson S, Illambas J, Siedek E, Thomas A, King V, Stirling C, Plevová E, Salt J, Sture G (2017). The administration of a single dose of a multivalent (DHPPiL4R) vaccine prevents clinical signs and mortality following virulent challenge with canine distemper virus, canine adenovirus or canine parvovirus. Trials Vaccinol.

[25] Twark L, Dodds WJ (2000). Clinical use of serum parvovirus and distemper virus antibody titers for determining revaccination strategies in healthy dogs. J Am Vet Med Assoc.

[26] Sepúlveda N, Stresman G, White MT, Drakeley CJ (2015). Current mathematical models for analyzing anti-malarial antibody data with an eye to malaria elimination and eradication. J Immunol Res.

